# Dichlorido{4-cyclo­hexyl-1-[1-(2-pyridyl-κ*N*)ethyl­idene]thio­semicarbazidato-κ^2^ *N* ^1^,*S*}methyl­tin(IV)

**DOI:** 10.1107/S1600536810014443

**Published:** 2010-04-24

**Authors:** Md. Abdus Salam, Md. Abu Affan, Mustaffa Shamsuddin, Seik Weng Ng

**Affiliations:** a Faculty of Resource Science and Technology, Universiti Malaysia Sarawak, 94300 Kota Samarahan, Sarawak, Malaysia; b Department of Chemistry, University of Malaya, 50603 Kuala Lumpur, Malaysia

## Abstract

The monodeprotonated Schiff base ligand in the title compound, [Sn(CH_3_)(C_14_H_19_N_4_S)Cl_2_], *N*,*N*′,*S*-chelates to the Sn atom, which is six-coordinated in an octa­hedral environment. The three coordinating atoms along with the methyl C atom comprise a square plane, above and below which are positioned the Cl atoms. The amino group is a hydrogen-bond donor to a Cl atom of an adjacent mol­ecule, the hydrogen bond giving rise to a helical chain extending parallel to [100].

## Related literature

For the crystal structures of other metal derivatives of the Schiff base, see: Joseph *et al.* (2004 ▶); Kovala-Demertzi *et al.* (2007 ▶).
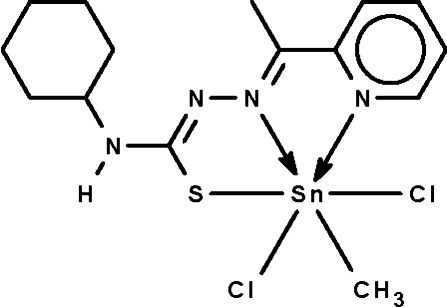


## Experimental

### 

#### Crystal data

[Sn(CH_3_)(C_14_H_19_N_4_S)Cl_2_]
*M* *_r_* = 480.02
Orthorhombic, 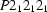

*a* = 9.2016 (5) Å
*b* = 12.2434 (7) Å
*c* = 17.4544 (10) Å
*V* = 1966.39 (19) Å^3^
*Z* = 4
Mo *K*α radiation
μ = 1.68 mm^−1^
*T* = 293 K
0.30 × 0.25 × 0.20 mm

#### Data collection

Bruker SMART APEX diffractometer
Absorption correction: multi-scan (*SADABS*; Sheldrick, 1996 ▶) *T*_min_ = 0.633, *T*_max_ = 0.730
18838 measured reflections
4498 independent reflections
4223 reflections with *I* > 2σ(*I*)
*R*_int_ = 0.025

#### Refinement

*R*[*F* ^2^ > 2σ(*F* ^2^)] = 0.021
*wR*(*F* ^2^) = 0.054
*S* = 1.02
4498 reflections
214 parameters
1 restraint
H atoms treated by a mixture of independent and constrained refinement
Δρ_max_ = 0.29 e Å^−3^
Δρ_min_ = −0.41 e Å^−3^
Absolute structure: Flack (1983 ▶), 1493 Friedel pairs
Flack parameter: −0.020 (17)

### 

Data collection: *APEX2* (Bruker, 2009 ▶); cell refinement: *SAINT* (Bruker, 2009 ▶); data reduction: *SAINT*; program(s) used to solve structure: *SHELXS97* (Sheldrick, 2008 ▶); program(s) used to refine structure: *SHELXL97* (Sheldrick, 2008 ▶); molecular graphics: *X-SEED* (Barbour, 2001 ▶); software used to prepare material for publication: *publCIF* (Westrip, 2010 ▶).

## Supplementary Material

Crystal structure: contains datablocks global, I. DOI: 10.1107/S1600536810014443/bt5250sup1.cif

Structure factors: contains datablocks I. DOI: 10.1107/S1600536810014443/bt5250Isup2.hkl

Additional supplementary materials:  crystallographic information; 3D view; checkCIF report

## Figures and Tables

**Table 1:** Selected bond lengths (Å)

Sn1—C1	2.136 (3)
Sn1—N1	2.269 (2)
Sn1—N2	2.224 (2)
Sn1—S1	2.4814 (7)
Sn1—Cl1	2.4960 (7)
Sn1—Cl2	2.4701 (8)

**Table 2:** Hydrogen-bond geometry (Å, °)

*D*—H⋯*A*	*D*—H	H⋯*A*	*D*⋯*A*	*D*—H⋯*A*
N4—H4⋯Cl1^i^	0.86 (1)	2.36 (1)	3.219 (3)	177 (3)

Symmetry code: (i) 
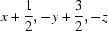
.

**Fig. 1.**
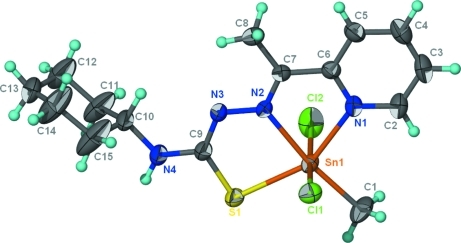
Anisotropic displacement ellipsoid plot (Barbour, 2001) of SnCl2(CH3)(C14H19N4S) at the 50% probability level; hydrogen atoms are drawn as spheres of arbitrary radius.

## supplementary crystallographic information

### Comment

The mono-deprotonated anion of 2-acetylpyridine 4-cyclohexyl thiosemicarbazone is a ligand that *N*,*N*',*S*-binds to metal atoms (Joseph *et al.*, 2004). Whereas similar ligands have been complexed with diorganotin and triorganotin systems, the monoorganotin analogues have not been so extensively studied. The mono-deprotonated Schiff-base ligand in SnCl_2_(CH_3_)(C_14_H_19_N_4_S) *N*,*N*',*S*-chelates to the tin atom, which is six-coordinate in an octahedral environment (Scheme I, Fig. 1). The three coordinating atoms along with the methyl carbon comprise a square plane, above and below which are positioned the chlorine atoms.

### Experimental

2-Acetylpyridine 4-cyclohexyl thiosemicarbazone was synthesized by using a literature method (Joseph *et al.*, 2004). The compound (0.28 g, 1 mmol) was dissolved in dry methanol (10 ml) in a Schlenk apparatus under a nitrogen atmosphere. Methyltin trichoride (0.24 g, 1 mmol) dissolved in methanol (10 ml) was added. The mixture was heated for an hour. The solvent was removed and the yellow compound recrystallized from chloroform/methanol (1:1) in 70% yield, m.p. 551-553 K.

### Refinement

Carbon-bound H-atoms were placed in calculated positions (C—H 0.93 to 0.978 Å) and were included in the refinement in the riding model approximation, with *U*(H) set to 1.2 to 1.5*U*(C).

The amino H-atom was located in a difference Fourier map, and was refined isotropically with a distance restraint of N–H 0.84±0.01 Å.

### Crystal data

**Table:**

[Sn(CH_3_)(C_14_H_19_N_4_S)Cl_2_]	*F*(000) = 960
*M**_r_* = 480.02	*D*_x_ = 1.621 Mg m^−^^3^
Orthorhombic, *P*2_1_2_1_2_1_	Mo *K*α radiation, λ = 0.71073 Å
Hall symbol: P 2ac 2ab	Cell parameters from 8827 reflections
*a* = 9.2016 (5) Å	θ = 2.3–27.7°
*b* = 12.2434 (7) Å	µ = 1.68 mm^−^^1^
*c* = 17.4544 (10) Å	*T* = 293 K
*V* = 1966.39 (19) Å^3^	Prism, yellow
*Z* = 4	0.30 × 0.25 × 0.20 mm

### Data collection

**Table:**

Bruker SMART APEX diffractometer	4498 independent reflections
Radiation source: fine-focus sealed tube	4223 reflections with *I* > 2σ(*I*)
graphite	*R*_int_ = 0.025
ω scans	θ_max_ = 27.5°, θ_min_ = 2.0°
Absorption correction: multi-scan (*SADABS*; Sheldrick, 1996)	*h* = −11→11
*T*_min_ = 0.633, *T*_max_ = 0.730	*k* = −15→15
18838 measured reflections	*l* = −22→22

### Refinement

**Table:**

Refinement on *F*^2^	Secondary atom site location: difference Fourier map
Least-squares matrix: full	Hydrogen site location: inferred from neighbouring sites
*R*[*F*^2^ > 2σ(*F*^2^)] = 0.021	H atoms treated by a mixture of independent and constrained refinement
*wR*(*F*^2^) = 0.054	*w* = 1/[σ^2^(*F*_o_^2^) + (0.032*P*)^2^ + 0.1524*P*] where *P* = (*F*_o_^2^ + 2*F*_c_^2^)/3
*S* = 1.02	(Δ/σ)_max_ = 0.001
4498 reflections	Δρ_max_ = 0.29 e Å^−^^3^
214 parameters	Δρ_min_ = −0.41 e Å^−^^3^
1 restraint	Absolute structure: Flack (1983), 1493 Friedel pairs
Primary atom site location: structure-invariant direct methods	Flack parameter: −0.020 (17)

### Fractional atomic coordinates and isotropic or equivalent isotropic displacement parameters (Å^2^)

**Table:**

	*x*	*y*	*z*	*U*_iso_*/*U*_eq_	
Sn1	1.061930 (18)	0.634199 (13)	0.160119 (10)	0.03748 (6)	
Cl1	0.97585 (8)	0.54078 (6)	0.04139 (4)	0.04922 (17)	
Cl2	1.10680 (12)	0.73379 (8)	0.28090 (5)	0.0724 (3)	
S1	1.22890 (7)	0.75142 (6)	0.08461 (5)	0.04529 (16)	
N1	0.8388 (3)	0.59846 (18)	0.20943 (13)	0.0411 (5)	
N2	0.9157 (2)	0.77150 (16)	0.12825 (11)	0.0351 (5)	
N3	0.9636 (2)	0.85860 (18)	0.08811 (12)	0.0398 (5)	
N4	1.1533 (3)	0.9409 (2)	0.02881 (19)	0.0602 (7)	
H4	1.2404 (17)	0.944 (3)	0.0111 (18)	0.063 (11)*	
C1	1.1749 (4)	0.4916 (3)	0.1978 (2)	0.0687 (10)	
H1A	1.1755	0.4893	0.2527	0.103*	
H1B	1.2731	0.4936	0.1792	0.103*	
H1C	1.1270	0.4277	0.1783	0.103*	
C2	0.8073 (4)	0.5114 (2)	0.25205 (17)	0.0524 (7)	
H2	0.8783	0.4586	0.2602	0.063*	
C3	0.6713 (4)	0.4978 (3)	0.28448 (19)	0.0599 (9)	
H3	0.6513	0.4370	0.3146	0.072*	
C4	0.5676 (4)	0.5746 (3)	0.27171 (17)	0.0589 (8)	
H4A	0.4761	0.5675	0.2938	0.071*	
C5	0.5985 (3)	0.6628 (2)	0.22600 (16)	0.0490 (7)	
H5	0.5271	0.7146	0.2157	0.059*	
C6	0.7362 (3)	0.6743 (2)	0.19527 (14)	0.0372 (6)	
C7	0.7802 (3)	0.7679 (2)	0.14765 (14)	0.0363 (5)	
C8	0.6739 (3)	0.8530 (3)	0.12494 (18)	0.0517 (7)	
H8A	0.7240	0.9121	0.1002	0.077*	
H8B	0.6249	0.8799	0.1697	0.077*	
H8C	0.6042	0.8222	0.0902	0.077*	
C9	1.1021 (3)	0.8555 (2)	0.06741 (14)	0.0400 (6)	
C10	1.0719 (4)	1.0403 (2)	0.0096 (2)	0.0595 (8)	
H10	0.9725	1.0328	0.0290	0.071*	
C11	1.1403 (6)	1.1369 (3)	0.0467 (2)	0.0941 (16)	
H11A	1.2421	1.1404	0.0324	0.113*	
H11B	1.1351	1.1288	0.1019	0.113*	
C12	1.0651 (7)	1.2431 (3)	0.0232 (2)	0.0936 (15)	
H12A	0.9671	1.2436	0.0437	0.112*	
H12B	1.1171	1.3044	0.0452	0.112*	
C13	1.0586 (6)	1.2563 (3)	−0.0593 (2)	0.0750 (11)	
H13A	1.1561	1.2688	−0.0786	0.090*	
H13B	1.0007	1.3203	−0.0712	0.090*	
C14	0.9968 (8)	1.1623 (3)	−0.0982 (3)	0.123 (2)	
H14A	1.0086	1.1717	−0.1531	0.147*	
H14B	0.8935	1.1592	−0.0875	0.147*	
C15	1.0660 (8)	1.0544 (3)	−0.0745 (2)	0.115 (2)	
H15A	1.0109	0.9947	−0.0967	0.138*	
H15B	1.1639	1.0509	−0.0950	0.138*	

### Atomic displacement parameters (Å^2^)

**Table:**

	*U*^11^	*U*^22^	*U*^33^	*U*^12^	*U*^13^	*U*^23^
Sn1	0.03555 (9)	0.03065 (8)	0.04623 (9)	0.00216 (7)	−0.00566 (7)	0.00345 (7)
Cl1	0.0458 (4)	0.0469 (4)	0.0550 (4)	0.0002 (3)	−0.0045 (3)	−0.0088 (3)
Cl2	0.0986 (7)	0.0686 (5)	0.0500 (4)	−0.0184 (5)	−0.0149 (4)	−0.0065 (4)
S1	0.0341 (3)	0.0362 (3)	0.0656 (4)	0.0016 (3)	0.0043 (3)	0.0044 (3)
N1	0.0436 (13)	0.0366 (11)	0.0431 (11)	−0.0039 (10)	−0.0010 (9)	0.0059 (9)
N2	0.0395 (12)	0.0274 (10)	0.0383 (10)	0.0034 (9)	0.0030 (9)	0.0019 (8)
N3	0.0371 (11)	0.0332 (10)	0.0491 (11)	0.0028 (10)	0.0050 (9)	0.0071 (10)
N4	0.0442 (15)	0.0431 (14)	0.093 (2)	0.0067 (11)	0.0179 (14)	0.0239 (14)
C1	0.060 (2)	0.0515 (19)	0.095 (3)	0.0150 (17)	−0.021 (2)	0.0203 (18)
C2	0.061 (2)	0.0445 (16)	0.0518 (16)	−0.0074 (15)	−0.0060 (15)	0.0125 (13)
C3	0.067 (2)	0.058 (2)	0.0554 (17)	−0.0250 (18)	−0.0023 (16)	0.0152 (15)
C4	0.0554 (18)	0.067 (2)	0.0543 (16)	−0.0200 (19)	0.0087 (16)	0.0020 (14)
C5	0.0451 (17)	0.0505 (17)	0.0513 (15)	−0.0044 (12)	0.0106 (13)	−0.0029 (12)
C6	0.0389 (14)	0.0353 (13)	0.0375 (12)	−0.0017 (11)	0.0023 (11)	−0.0067 (10)
C7	0.0371 (13)	0.0348 (12)	0.0371 (13)	0.0036 (10)	0.0032 (10)	−0.0028 (10)
C8	0.0415 (15)	0.0502 (17)	0.0632 (16)	0.0141 (14)	0.0045 (13)	0.0060 (14)
C9	0.0426 (14)	0.0317 (13)	0.0458 (13)	−0.0002 (12)	0.0011 (10)	0.0017 (11)
C10	0.0435 (15)	0.0419 (15)	0.093 (2)	0.0065 (15)	0.0162 (18)	0.0256 (15)
C11	0.159 (5)	0.059 (2)	0.064 (2)	0.046 (3)	−0.036 (3)	−0.0167 (19)
C12	0.154 (4)	0.052 (2)	0.075 (2)	0.032 (3)	−0.021 (3)	−0.0103 (19)
C13	0.092 (3)	0.0485 (18)	0.085 (2)	0.013 (2)	0.013 (2)	0.0252 (17)
C14	0.214 (7)	0.071 (3)	0.083 (3)	0.057 (3)	−0.064 (4)	−0.008 (2)
C15	0.197 (6)	0.058 (2)	0.090 (3)	0.049 (3)	−0.067 (4)	−0.022 (2)

### Geometric parameters (Å, °)

**Table:**

Sn1—C1	2.136 (3)	C5—C6	1.383 (4)
Sn1—N1	2.269 (2)	C5—H5	0.9300
Sn1—N2	2.224 (2)	C6—C7	1.473 (4)
Sn1—S1	2.4814 (7)	C7—C8	1.483 (4)
Sn1—Cl1	2.4960 (7)	C8—H8A	0.9600
Sn1—Cl2	2.4701 (8)	C8—H8B	0.9600
S1—C9	1.753 (3)	C8—H8C	0.9600
N1—C2	1.332 (4)	C10—C15	1.479 (5)
N1—C6	1.348 (3)	C10—C11	1.488 (6)
N2—C7	1.292 (3)	C10—H10	0.9800
N2—N3	1.350 (3)	C11—C12	1.529 (5)
N3—C9	1.326 (3)	C11—H11A	0.9700
N4—C9	1.330 (4)	C11—H11B	0.9700
N4—C10	1.468 (4)	C12—C13	1.450 (5)
N4—H4	0.861 (10)	C12—H12A	0.9700
C1—H1A	0.9600	C12—H12B	0.9700
C1—H1B	0.9600	C13—C14	1.453 (6)
C1—H1C	0.9600	C13—H13A	0.9700
C2—C3	1.383 (5)	C13—H13B	0.9700
C2—H2	0.9300	C14—C15	1.523 (5)
C3—C4	1.359 (5)	C14—H14A	0.9700
C3—H3	0.9300	C14—H14B	0.9700
C4—C5	1.372 (4)	C15—H15A	0.9700
C4—H4A	0.9300	C15—H15B	0.9700
			
C1—Sn1—N2	171.66 (12)	N2—C7—C8	122.8 (2)
C1—Sn1—N1	99.56 (12)	C6—C7—C8	121.1 (2)
N2—Sn1—N1	72.12 (8)	C7—C8—H8A	109.5
C1—Sn1—Cl2	93.43 (11)	C7—C8—H8B	109.5
N2—Sn1—Cl2	86.65 (6)	H8A—C8—H8B	109.5
N1—Sn1—Cl2	85.57 (6)	C7—C8—H8C	109.5
C1—Sn1—S1	109.56 (11)	H8A—C8—H8C	109.5
N2—Sn1—S1	78.74 (6)	H8B—C8—H8C	109.5
N1—Sn1—S1	150.85 (6)	N3—C9—N4	117.1 (3)
Cl2—Sn1—S1	93.69 (3)	N3—C9—S1	127.9 (2)
C1—Sn1—Cl1	92.01 (11)	N4—C9—S1	115.0 (2)
N2—Sn1—Cl1	86.94 (6)	N4—C10—C15	110.0 (3)
N1—Sn1—Cl1	86.53 (6)	N4—C10—C11	110.1 (3)
Cl2—Sn1—Cl1	171.05 (3)	C15—C10—C11	110.8 (3)
S1—Sn1—Cl1	91.18 (3)	N4—C10—H10	108.6
C9—S1—Sn1	95.70 (9)	C15—C10—H10	108.6
C2—N1—C6	120.1 (3)	C11—C10—H10	108.6
C2—N1—Sn1	124.3 (2)	C10—C11—C12	111.5 (3)
C6—N1—Sn1	115.58 (17)	C10—C11—H11A	109.3
C7—N2—N3	118.5 (2)	C12—C11—H11A	109.3
C7—N2—Sn1	119.51 (17)	C10—C11—H11B	109.3
N3—N2—Sn1	121.94 (16)	C12—C11—H11B	109.3
C9—N3—N2	115.7 (2)	H11A—C11—H11B	108.0
C9—N4—C10	126.0 (3)	C13—C12—C11	112.3 (3)
C9—N4—H4	123 (2)	C13—C12—H12A	109.1
C10—N4—H4	111 (2)	C11—C12—H12A	109.1
Sn1—C1—H1A	109.5	C13—C12—H12B	109.1
Sn1—C1—H1B	109.5	C11—C12—H12B	109.1
H1A—C1—H1B	109.5	H12A—C12—H12B	107.9
Sn1—C1—H1C	109.5	C12—C13—C14	113.1 (4)
H1A—C1—H1C	109.5	C12—C13—H13A	109.0
H1B—C1—H1C	109.5	C14—C13—H13A	109.0
N1—C2—C3	121.5 (3)	C12—C13—H13B	109.0
N1—C2—H2	119.3	C14—C13—H13B	109.0
C3—C2—H2	119.3	H13A—C13—H13B	107.8
C4—C3—C2	119.0 (3)	C13—C14—C15	113.3 (4)
C4—C3—H3	120.5	C13—C14—H14A	108.9
C2—C3—H3	120.5	C15—C14—H14A	108.9
C3—C4—C5	119.6 (3)	C13—C14—H14B	108.9
C3—C4—H4A	120.2	C15—C14—H14B	108.9
C5—C4—H4A	120.2	H14A—C14—H14B	107.7
C4—C5—C6	119.7 (3)	C10—C15—C14	112.7 (4)
C4—C5—H5	120.1	C10—C15—H15A	109.0
C6—C5—H5	120.1	C14—C15—H15A	109.0
N1—C6—C5	120.1 (2)	C10—C15—H15B	109.0
N1—C6—C7	116.6 (2)	C14—C15—H15B	109.0
C5—C6—C7	123.4 (2)	H15A—C15—H15B	107.8
N2—C7—C6	116.1 (2)		
			
C1—Sn1—S1—C9	−178.73 (15)	C3—C4—C5—C6	−1.9 (5)
N2—Sn1—S1—C9	2.07 (10)	C2—N1—C6—C5	1.0 (4)
N1—Sn1—S1—C9	3.74 (16)	Sn1—N1—C6—C5	−176.8 (2)
Cl2—Sn1—S1—C9	−83.77 (9)	C2—N1—C6—C7	180.0 (2)
Cl1—Sn1—S1—C9	88.72 (9)	Sn1—N1—C6—C7	2.1 (3)
C1—Sn1—N1—C2	2.9 (3)	C4—C5—C6—N1	0.8 (4)
N2—Sn1—N1—C2	−177.8 (2)	C4—C5—C6—C7	−178.0 (3)
Cl2—Sn1—N1—C2	−89.9 (2)	N3—N2—C7—C6	−176.5 (2)
S1—Sn1—N1—C2	−179.50 (18)	Sn1—N2—C7—C6	4.4 (3)
Cl1—Sn1—N1—C2	94.3 (2)	N3—N2—C7—C8	2.6 (4)
C1—Sn1—N1—C6	−179.4 (2)	Sn1—N2—C7—C8	−176.5 (2)
N2—Sn1—N1—C6	−0.01 (17)	N1—C6—C7—N2	−4.3 (3)
Cl2—Sn1—N1—C6	87.89 (18)	C5—C6—C7—N2	174.6 (2)
S1—Sn1—N1—C6	−1.7 (3)	N1—C6—C7—C8	176.6 (2)
Cl1—Sn1—N1—C6	−87.92 (18)	C5—C6—C7—C8	−4.5 (4)
C1—Sn1—N2—C7	1.9 (9)	N2—N3—C9—N4	−179.0 (3)
N1—Sn1—N2—C7	−2.46 (18)	N2—N3—C9—S1	1.1 (3)
Cl2—Sn1—N2—C7	−88.88 (19)	C10—N4—C9—N3	2.6 (5)
S1—Sn1—N2—C7	176.68 (19)	C10—N4—C9—S1	−177.5 (3)
Cl1—Sn1—N2—C7	84.87 (19)	Sn1—S1—C9—N3	−2.6 (3)
C1—Sn1—N2—N3	−177.3 (8)	Sn1—S1—C9—N4	177.5 (2)
N1—Sn1—N2—N3	178.4 (2)	C9—N4—C10—C15	−120.0 (5)
Cl2—Sn1—N2—N3	91.99 (17)	C9—N4—C10—C11	117.6 (4)
S1—Sn1—N2—N3	−2.45 (17)	N4—C10—C11—C12	175.8 (4)
Cl1—Sn1—N2—N3	−94.26 (17)	C15—C10—C11—C12	53.9 (6)
C7—N2—N3—C9	−177.5 (2)	C10—C11—C12—C13	−54.4 (7)
Sn1—N2—N3—C9	1.6 (3)	C11—C12—C13—C14	52.4 (7)
C6—N1—C2—C3	−1.8 (4)	C12—C13—C14—C15	−50.4 (8)
Sn1—N1—C2—C3	175.8 (2)	N4—C10—C15—C14	−174.0 (5)
N1—C2—C3—C4	0.7 (5)	C11—C10—C15—C14	−52.0 (7)
C2—C3—C4—C5	1.2 (5)	C13—C14—C15—C10	50.5 (8)

### Hydrogen-bond geometry (Å, °)

**Table:**

*D*—H···*A*	*D*—H	H···*A*	*D*···*A*	*D*—H···*A*
N4—H4···Cl1^i^	0.86 (1)	2.36 (1)	3.219 (3)	177 (3)

Symmetry codes: (i) *x*+1/2, −*y*+3/2, −*z*.
