# Dichlorido{4-cyclo­hexyl-1-[1-(2-pyridyl-κ*N*)ethyl­idene]thio­semicarbazidato-κ^2^ *N* ^1^,*S*}methyl­tin(IV). Corrigendum

**DOI:** 10.1107/S1600536810049214

**Published:** 2010-12-18

**Authors:** Md. Abdus Salam, Md. Abu Affan, Mustaffa Shamsuddin, Seik Weng Ng

**Affiliations:** a Faculty of Resource Science and Technology, Universiti Malaysia Sarawak, 94300 Kota Samarahan, Sarawak, Malaysia; b Department of Chemistry, Faculty of Science, Universiti Teknologi Malaysia, 81310 UTM Skudai, Johor, Malaysia; c Depart­ment of Chemistry, University of Malaya, 50603 Kuala Lumpur, Malaysia

## Abstract

The address of one of the authors in the paper by Salam *et al.* [*Acta Cryst.* (2010), E**66**, m570] is corrected.

## Abstract 2

Corrigendum to *Acta Cryst.* (2010), E**66**, m570.

In the paper by Salam *et al.* (2010) ▶, the address of Mustaffa Shamsuddin is given incorrectly. The correct address is ‘Department of Chemistry, Faculty of Science, Universiti Teknologi Malaysia, 81310 UTM Skudai, Johor, Malaysia’, as given above.
